# Markers of Hemophagocytic Lymphohistiocytosis Are Associated with Mortality in Critically Ill Patients

**DOI:** 10.3390/jcm14061970

**Published:** 2025-03-14

**Authors:** Max Lenz, Patrick Haider, Eva Steinacher, Constantin Gatterer, Robert Zilberszac, Svitlana Demyanets, Christian Hengstenberg, Johann Wojta, Gottfried Heinz, Walter S. Speidl, Konstantin A. Krychtiuk

**Affiliations:** 1 Department of Internal Medicine II, Division of Cardiology, Medical University of Vienna, 1090 Vienna, Austria; max.lenz@meduniwien.ac.at (M.L.); patrick.haider@meduniwien.ac.at (P.H.); eva.steinacher@meduniwien.ac.at (E.S.); constantin.gatterer@meduniwien.ac.at (C.G.); robert.zilberszac@meduniwien.ac.at (R.Z.); christian.hengstenberg@meduniwien.ac.at (C.H.); johann.wojta@meduniwien.ac.at (J.W.); gottfried.heinz@meduniwien.ac.at (G.H.); konstantin.krychtiuk@meduniwien.ac.at (K.A.K.); 2 Ludwig Boltzmann Institute for Cardiovascular Research, 1090 Vienna, Austria; 3 Department of Laboratory Medicine, Klinik Hietzing, 1130 Vienna, Austria; svitlana.demyanets@meduniwien.ac.at; 4 Department of Laboratory Medicine, Medical University of Vienna, 1090 Vienna, Austria; 5 Core Facilities, Medical University of Vienna, 1090 Vienna, Austria

**Keywords:** critical care, hemophagocytic lymphohistiocytosis, CD25, hemophagocytosis

## Abstract

**Background:** Critically ill patients often display systemic immune dysregulation and increased inflammatory activity. Hemophagocytic lymphohistiocytosis (HLH) represents a rare syndrome defined by the inappropriate survival of cytotoxic T cells and the occurrence of cytokine storms. Although HLH is characterized by relatively high mortality rates, little is known about the predictive value of its diagnostic criteria. Accordingly, our objective was to evaluate these properties within an unselected cohort of critically ill patients admitted to a tertiary intensive care unit (ICU). **Methods:** This single-center prospective observational study included 176 consecutive patients. Available HLH criteria at admission were assessed, including sCD25 measurements performed using ELISA. **Results:** Overall, 30-day mortality rates were significantly higher in patients exhibiting two or more criteria of HLH (21.9% vs. 43.3%, *p* = 0.033). Moreover, sCD25 emerged as an independent risk predictor of 30-day mortality independent of age, sex, the use of vasopressors, and mechanical ventilation (HR 2.72 for the highest tertile vs. lowest tertile, *p* = 0.012). Additionally, fibrinogen was significantly decreased in non-survivors (*p* = 0.019), and its addition to the SAPS II score significantly increased its prognostic capability (*p* = 0.045). In contrast, ferritin and triglyceride levels were not different in survivors versus non-survivors. **Conclusions:** Critically ill patients displaying two or more HLH criteria exhibit a dramatic increase in 30-day mortality, even in the absence of an established HLH diagnosis. Furthermore, elevated levels of sCD25 and decreased levels of fibrinogen were found to be significant predictors of mortality.

## 1. Introduction

Critically ill patients admitted to the intensive care unit (ICU) display a diverse range of underlying illnesses and critical medical conditions. Despite the heterogeneity of the disease, patients often show systemic immune dysregulation paired with significantly increased inflammatory activity [1]. Hemophagocytic lymphohistiocytosis (HLH) represents a rare syndrome characterized by fever, inflammation, cytopenia, and organ dysfunction. The inappropriate survival of cytotoxic T cells as well as histiocytes triggers cytokine storms, accompanied by the occurrence of hemophagocytosis and multi-organ failure [2]. In addition to familial forms, secondary HLH can be caused by active malignancy, autoimmune dysregulation, and severe infection [3]. Due to a similar presentation, acute states of inflammation such as sepsis, systemic inflammatory response syndrome (SIRS), and HLH can coexist and overlap with each other, hampering early detection as well as correct diagnosis and potentially delaying treatment [4].

The fact that conventional criteria for secondary HLH are not thoroughly validated for critically ill patients requiring ICU admission makes the adequate detection of cases even more challenging [5,6]. Thus, the prevalence of HLH in critical illness likely remains underreported [7]. One sizeable retrospective analysis within the U.S. Nationwide Inpatient Sample included all admissions from about 1000 hospitals within eight years. It revealed that around 30% of the 7400 estimated discharges with HLH met the criteria for critical illness. Notably, almost half of these individuals died during their stay at the hospital [8]. Therefore, the aim of this single-center, prospective study was to evaluate the degree to which HLH criteria were met in an unselected, consecutively enrolled critically ill patient population admitted to a tertiary cardiac ICU and assess their association with 30-day mortality.

## 2. Materials and Methods

### 2.1. Study Design and Population

This was a prospective observational study conducted at the medical ICU of the Division of Cardiology, Department of Internal Medicine II at the Vienna General Hospital [9,10]. Our tertiary ICU treats a heterogeneous population of critically ill patients, with a particular emphasis on acute cardiovascular conditions. To ensure an unbiased study cohort, all admitted patients were systematically enrolled after providing written informed consent. The institutional ethics board waived the requirement for informed consent in unconscious individuals. Only patients aged 18 years and older were included in the analysis. Additionally, we excluded individuals with active human immunodeficiency virus (HIV) or hepatitis C virus (HCV) infections. The study adhered to the principles of the Declaration of Helsinki for biomedical research involving human subjects and complied with ICH Good Clinical Practice Guidelines. The local ethics committee of the Medical University of Vienna approved the study protocol (EK 1101/2012). A total of 233 patients were enrolled, with HLH criteria data available for 176 participants. The primary study outcome was 30-day mortality, and all patients were monitored for this endpoint.

### 2.2. Data Acquisition and Blood Sampling

Baseline demographics, primary admission diagnosis, medical history, and routine laboratory parameters were recorded within 24 h of ICU admission. Furthermore, information on major interventions occurring within the first 72 h of admission was gathered. These interventions encompassed mechanical ventilation, renal replacement therapy, major surgical procedures, and the administration of catecholamines or extracorporeal membrane oxygenation (ECMO). Additionally, all patients underwent screening for systemic inflammatory response syndrome (SIRS) criteria. The severity of illness and associated mortality risk were assessed using the Simplified Acute Physiology Score II (SAPS II) and the Sequential Organ Failure Assessment (SOFA) score. Blood samples were obtained upon admission using an arterial catheter. If an arterial line was unavailable, samples were collected via a central venous catheter. To maintain sample integrity, 3 mL of blood was initially discarded before collection. Routine laboratory analyses followed local standards established by the Department of Laboratory Medicine at the Medical University of Vienna. Additionally, one EDTA tube, one 3.8% sodium citrate tube, and one serum separator tube (all Greiner Bio-One, Kremsmünster, Austria) were centrifuged at 4 °C at 3000 RPM and subsequently stored at −80 °C for future analyses [11,12].

### 2.3. Assessment of HLH Criteria

This study was conducted prospectively, with all necessary source data collected in paper form at admission and during the first 72 h. After the enrollment phase was completed, the database was finalized, and available HLH criteria were systematically entered. The diagnosis of HLH is based on the presence of defined criteria, as described in the literature (HLH-2004 criteria) [13,14]. Accordingly, the presence of HLH is defined by fulfilling five out of the eight criteria (Supplementary Table S1). Fever was defined as a peak temperature exceeding 38.5 °C (101.3 °F) within the first 24 h of ICU admission, as recorded in the electronic health record. Splenomegaly was determined via radiological imaging, with patients undergoing either computed tomography (CT) scanning or abdominal ultrasound. For CT imaging, a craniocaudal spleen length exceeding 10 cm was indicative of splenomegaly. For ultrasound assessment, splenic enlargement was defined as ≥13 cm (superior-inferior axis), >6 cm (anterior–posterior), or >7 cm (medial-lateral axis) [15]. The prevalence of splenomegaly in unselected populations has been reported to range between 0.3% and 3% [16,17]. In our cohort, the prevalence was 4%, suggesting that the underestimation of splenomegaly was unlikely. Standard laboratory tests were used to determine platelet count, hemoglobin concentration, neutrophil count, triglyceride levels, fibrinogen, and serum ferritin [10,12,18]. Due to ethical considerations, bone marrow biopsy for the detection of hemophagocytosis was not routinely performed. Furthermore, technical limitations prevented the in vivo assessment of low or absent NK cell activity, making this criterion unavailable for our analysis. The measurement of soluble CD25 (sCD25) was conducted using an enzyme-linked immunosorbent assay (ELISA) following the manufacturer’s guidelines (Human CD25/IL-2R alpha, R&D Systems, Minnneapolis, MN, USA).

### 2.4. Statistical Analysis

Considering a mortality rate of approximately 25% in our cohort (with estimated rates of 21.5% in the 0–1 HLH criterion group and 43.0% in the 2+ criteria group, equating to a doubling in mortality), sample size calculations indicated that at least 170 patients were needed to detect this difference, with a power of 0.9 and a significance level of 0.05. With 176 patients included, our study appears sufficiently powered to detect meaningful differences between these groups. Continuous variables are presented as the median and interquartile range (IQR), and Levene’s test was utilized to determine the equality of variances. For categorical variables, counts or percentages were provided and compared using either the Chi-square or Fisher’s exact test, as appropriate. The Kolmogorov–Smirnov test was used to distinguish between parametric and non-parametric data. Non-parametric data were analyzed using the Mann–Whitney test, while the unpaired Student’s *t*-test was used for parametric data. ANOVA with Bonferroni correction was utilized to compare multiple groups. Kaplan–Meier survival curves were generated to depict the association between HLH criteria and 30-day mortality, with statistical significance assessed using the log-rank test. Cox proportional hazard regression was utilized to evaluate the prognostic significance of sCD25 and HLH criteria for 30-day mortality, adjusting for demographic variables, sepsis, vasopressor use, and mechanical ventilation. Receiver operating characteristic (ROC) curves were generated for 30-day survival, incorporating HLH criteria and established clinical scores as test variables. Combined predictive values were assessed using logistic regression models, and ROC curve comparisons were conducted as previously described [19]. Statistical analyses were performed using SPSS Statistics 27.0 (IBM Corporation, North Castle, NY, USA), R (The R Foundation for Statistical Computing, Vienna, Austria), and MedCalc Statistical Software 22.0 (MedCalc Software, Ostend, Belgium). Unless otherwise stated, a *p*-value < 0.05 was considered statistically significant.

## 3. Results

Of 233 consecutively admitted patients, 176 had complete information in terms of the defined HLH criteria and thus comprised our study population. Table 1 presents demographic data and baseline clinical characteristics. In total, 59 patients (33.5%) underwent cardiac surgery or interventional valve procedures and were admitted for post-operative/post-interventional observation. The remaining 117 patients (66.5%) were admitted for critical medical illness, with cardiac arrest (CA, 22.7%) and cardiogenic shock (CS, 14.2%) being the leading causes of ICU admission. The median age among all patients was 67.5 (IQR 57.5–77.4) years and 38.6% of patients were female. Overall, 30-day mortality was 25.6%, with a median SAPS II score of 45 (IQR 31–59) and a SOFA score of 8 (IQR 5–11), reflecting a severely ill collective of patients. Patients who died within 30 days after ICU admission more often required mechanical ventilation as well as vasopressors and exhibited higher lactate levels (Table 1, *p* = 0.004 for vasopressors, *p* = 0.025 for mechanical ventilation, and *p* = 0.012 for lactate levels). Furthermore, they exhibited increased serum creatinine and lower arterial pH levels compared to 30-day survivors (Table 1, *p* < 0.001 for serum creatinine, and *p* = 0.036 for low arterial pH).

**Figure 1:**
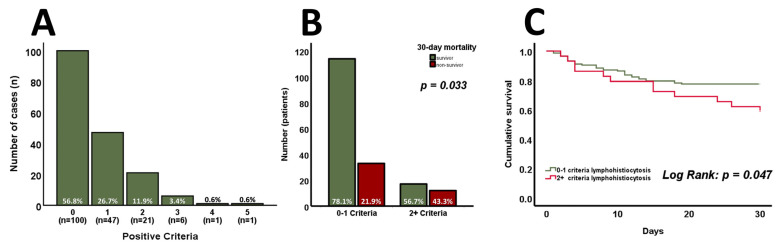
The distribution of positive HLH criteria at admission (*n* = 176) is shown in (**A**). Patients with zero to one positive criterion exhibit significantly lower 30-day mortality and cumulative survival compared to those with two or more positive criteria ((**B**,**C**), *n* = 176). *p*-values of <0.05 are considered statistically significant (Chi-square, Mann–Whitney test, unpaired Student’s *t*-test, and log-rank test were used).

The distribution of positive HLH criteria at admission is shown in Figure 1A. Only 1 out of the 176 patients had definite HLH with five positive criteria. However, it must be emphasized that two out of eight criteria were unavailable as bone marrow aspiration and the measurement of NK cell activity were not routinely performed. Patients were further categorized into two groups based on HLH criteria: those with no or one positive criterion (*n* = 147; 83.5%) and those with two or more criteria (*n* = 29; 16.5%). The 30-day mortality rate for patients with zero to one positive criterion was 21.9%, compared to 43.3% in the group with two or more criteria (Figure 1B, *p* = 0.033). Moreover, cumulative survival significantly differed between the two groups (Figure 1C, log-rank *p* = 0.047). A detailed comparison of the groups’ baseline characteristics can be found in Table 2. Patients fulfilling two or more criteria were significantly younger (*p* = 0.031) and exhibited lower levels of hemoglobin (*p* = 0.001) as well as platelet counts (*p* < 0.001). Moreover, they displayed increased CRP (*p* = 0.003), creatinine (*p* = 0.006), and bilirubin (*p* = 0.001) levels. Interestingly, no tangible differences in the SAPS II score were found at admission (*p* = 0.166). In contrast, the SOFA score significantly increased (*p* = 0.001) in patients with two or more HLH criteria. Such patients were less frequently admitted due to observation following cardiac surgery or valve interventions (*p* = 0.004), but more often exhibited sepsis and septic shock as causes of admission (all Table 2, *p* = 0.017).

**Figure 2:**
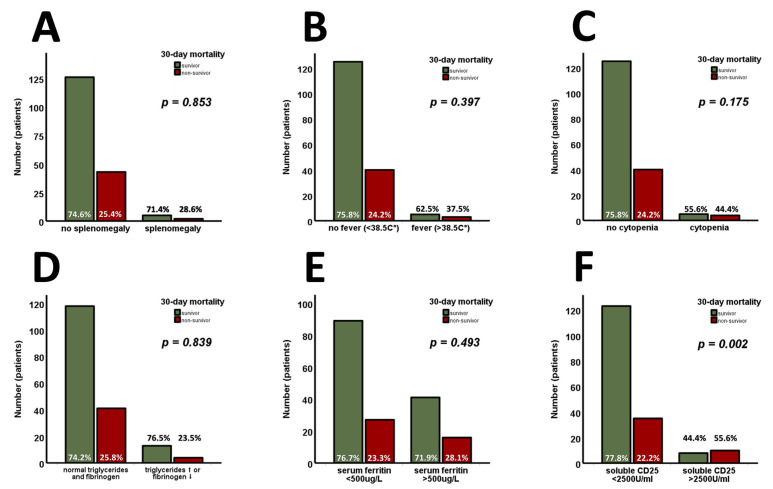
The available criteria for HLH are analyzed in the entire cohort (*n* = 176). Splenomegaly (**A**), fever (**B**), cytopenia (**C**), triglycerides/fibrinogen (**D**), and serum ferritin (**E**) are found without prognostic properties for 30-day survival. Only sCD25 (>2500 U/mL) is found to be significantly higher at admission in 30-day non-survivors (**F**). *p*-values of <0.05 are considered statistically significant (Chi-square, Mann–Whitney test, and unpaired Student’s *t*-test were used). sCD25—the soluble cluster of differentiation 25.

**Figure 3:**
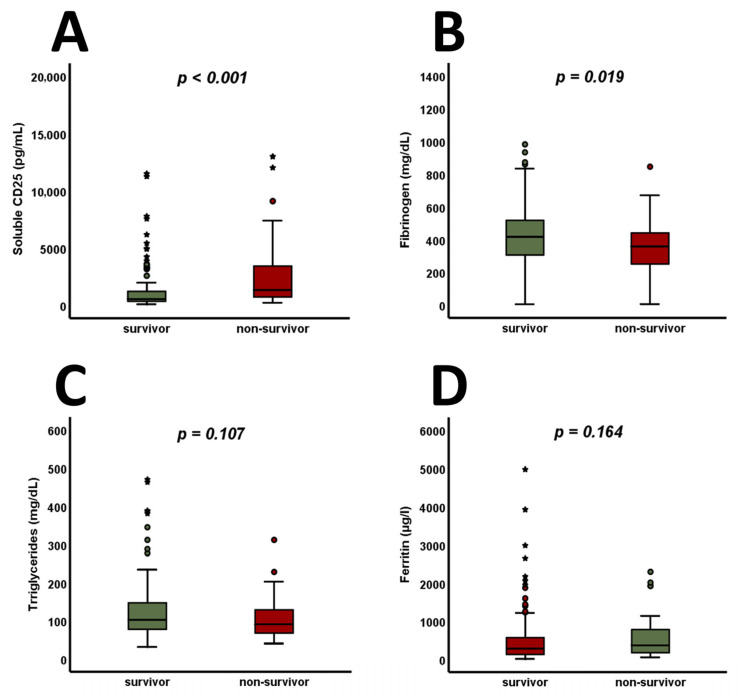
In 30-day non-survivors, sCD25 is found to increase (**A**), whereas fibrinogen is found to decrease (**B**). At admission, no significant changes are observable when analyzing triglyceride (**C**) and ferritin (**D**) levels. *p*-values of <0.05 are considered statistically significant (Mann–Whitney test and unpaired Student’s *t*-test were used). Circles indicate extreme values, whereas stars mark outliers. CD25—cluster of differentiation 25.

The available criteria for HLH were analyzed in the entire cohort and are shown in Figure 2. Among these parameters, only sCD25 (>2500 U/mL) was found to be significantly higher at admission in 30-day non-survivors (Figure 2F, *p* = 0.002). Analyzing the continuous variables involved independently of their predefined cut-offs for HLH, sCD25 (Figure 3A, *p* < 0.001) was found to be increased, whereas fibrinogen (Figure 3B, *p* = 0.019) was found to be decreased, in 30-day non-survivors. Admission levels of triglycerides (Figure 3C, *p* = 0.107) and ferritin (Figure 3D, *p* = 0.164) did not differ between 30-day survivors and non-survivors.

**Figure 4:**
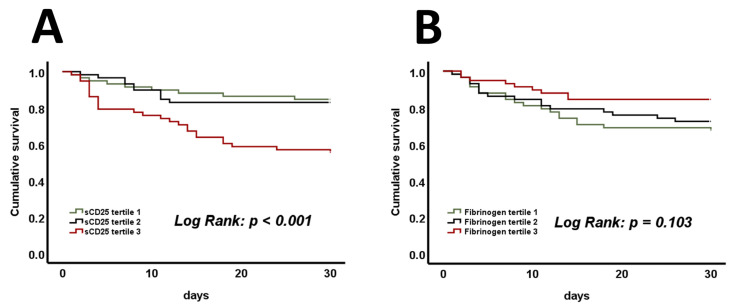
Significant differences in mortality are present when comparing the cumulative survival of sCD25 tertiles during admission to the ICU (**A**). No such differences are present when comparing tertiles of fibrinogen (**B**). *p*-values of <0.05 are considered statistically significant (log-rank test was used). sCD25—a soluble cluster of differentiation 25.

Additionally, Kaplan–Meier analysis assessed the prognostic abilities of tertiles of sCD25, fibrinogen, triglycerides, and ferritin. In terms of cumulative survival, only tertiles of sCD25 displayed significant differences (Figure 4A, log-rank *p* < 0.001), whereas fibrinogen (Figure 4B, log-rank *p* = 0.103), triglycerides (Supplementary Figure S1A, log-rank *p* = 0.471), and ferritin (Supplementary Figure S1B, log-rank *p* = 0.524) were not found to be predictive. Cox proportional hazard regression analysis was conducted to investigate the prognostic value of sCD25 tertiles and HLH criteria at admission for 30-day mortality. In a univariate analysis, comparing the first to the third tertile of sCD25 resulted in a hazard ratio (HR) of 3.44 (Supplementary Table S2, 95% confidence interval (CI) 1.61–7.35, *p* = 0.001). In a corresponding multivariate analysis, this finding was proven independently of age, sex, vasopressors, and mechanical ventilation, with an HR of 2.72 (Supplementary Table S2, 95% CI 1.24–5.97, *p* = 0.012). Similar findings were made by comparing patients with 0–1 positive HLH criterion with those exhibiting 2 or more. The univariate analysis revealed an HR of 2.06 (Supplementary Table S2, 95% CI 1.08–3.92, *p* = 0.029), whereas the multivariate analysis resulted in an HR of 2.10, and this was independent of age, sex, and the use of vasopressors and mechanical ventilation (Supplementary Table S2, 95% CI 1.06–4.10, *p* = 0.033). Furthermore, adjustment for age, sex, sepsis, and mechanical ventilation in a multivariate model confirmed that HLH criteria remained the strongest predictor of mortality, even when accounting for the diagnosis of sepsis (Supplementary Table S2, HR 2.07, 95% CI 1.06–4.02, *p* = 0.033). ROC curves were calculated for sCD25, fibrinogen, and the SAPS II score. An area under the curve (AUC) of 0.79 was determined for the SAPS II score and an AUC of 0.69 was determined for sCD25 (Supplementary Figure S2A). Combining both parameters’ prognostic properties resulted in an AUC of 0.81 and showed a trend toward better predictions of mortality within 30 days (Supplementary Figure S2A, *p* = 0.099). Analysis of fibrinogen revealed an AUC of 0.62 in the prediction of 30-day survival (Supplementary Figure S2B, AUC of 0.38 for 30-day mortality). Interestingly, adding fibrinogen to the SAPS II score significantly increased its prognostic value (Supplementary Figure S2B, AUC 0.83, *p* = 0.045). No such increases were found when adding triglycerides or ferritin (Supplementary Figure S3A,B).

## 4. Discussion

Our study comprises three major findings. Firstly, we identified sCD25 as the only relevant predictor of survival amongst the individually assessed HLH criteria. Secondly, we demonstrated the prognostic properties of sCD25 as well as fibrinogen regarding 30-day mortality when analyzed irrespective of HLH cutoffs. Thirdly, we were able to show that patients with two or more HLH criteria at ICU admission suffered from significantly increased mortality rates, even in the absence of a formal HLH diagnosis.

In our analyses, patients were categorized using the HLH-2004 criteria instead of the HScore, as the latter does not include sCD25, which emerged as the strongest predictor in our setting. Among the individual diagnostic criteria, the literature reports hyperferritinemia and sCD25 as potentially bearing prognostic importance in the intensive care setting [20]. Particularly in patients with sepsis, circulating sCD25 was associated with clinical outcomes [21]. Although both ferritin and sCD25 might help with differentiation, applying HLH criteria for ICU patients appears challenging due to overlap with conditions like sepsis and SIRS, which share clinical and laboratory features. Originally developed for pediatric and hematologic contexts, these criteria, excluding sCD25, may not fully capture the complexities of critical illness. The dynamic ICU environment and variability in biomarker availability further limit their utility, highlighting the need for refined, ICU-specific adaptations. Within our study, sCD25 emerged as a strong predictor of survival independently of traditional ICU risk factors such as age, sex, and the need for vasopressors or mechanical ventilation. Moreover, low fibrinogen levels were linked to worse outcomes, and their addition to the SAPS II score significantly improved its prognostic capacity. In contrast, other variables included in the diagnostic of HLH, such as ferritin and triglycerides, were not associated with 30-day mortality. Our findings are consistent with previous research highlighting the diagnostic challenges and prognostic implications of HLH in critical illness [4,22]. Our study identified sCD25 as a crucial indicator of mortality in unselected patients, consistent with prior observations of poor outcomes in septic patients [21,23]. Considering the overrepresentation of sepsis as a cause of admission in patients who met two or more HLH criteria, there seems to be a plausible connection. Furthermore, the significantly increased CRP levels in these individuals underline this finding. However, inflammatory conditions, including HLH, sepsis, and SIRS, appear to overlap. In this regard, sCD25 might be viewed as a marker of rampant inflammation, and thus may not be specific for pathogen-associated immune modulation. Moreover, septic triggers are suspected to promote the onset of HLH, blurring the line between primary infection and subsequent inflammatory activation even further [24].

Besides having lower 30-day survival rates, patients with positivity for two or more criteria were statistically younger and exhibited higher SOFA scores. Accordingly, these patients displayed increased signs of organ dysfunction, including elevated levels of bilirubin and creatinine and decreased hemoglobin values and platelet counts. At first glance, differences in hemodynamics and the occurrence of shock might be suspected to be the underlying cause. Surprisingly, the two groups showed no noticeable differences in heart rate, mean blood pressure, mechanical ventilation, and the use of vasopressors. These findings render hemodynamic causes unlikely and may suggest an alternative mechanism of organ impairment. A hyperinflammatory state associated with cytokine storms and endothelial injury may represent one possible explanation [25,26]. For example, aging has been associated with an attenuation of cytokine release by monocytes upon inflammatory stimulation, which appears to be associated with an increased risk of developing infectious diseases in the elderly [27,28]. Therefore, younger patients seem to be characterized by the excessive activation of the immune system accompanied by severe organ dysfunction without the inevitable occurrence of shock or hemodynamical instability.

As an isolated factor, hypertriglyceridemia and/or hypofibrinogenemia showed no predictive value for 30-day survival. However, low fibrinogen levels were significantly linked to increased mortality. Moreover, adding fibrinogen to the SAPS II score resulted in a statistically significant improvement in predictive capability. While the increase observed was modest, even small gains in prognostic accuracy can be meaningful in ICU settings, where refined risk stratification informs clinical decision-making. Previous reports have highlighted the relationship between fibrinogen levels and ICU outcomes, particularly in sepsis-induced coagulopathy [29,30]. Additionally, fibrinogen levels are associated with bleeding risk in cardiac surgery [31]. Interestingly, we observed the significant underrepresentation of surgical cases and a high occurrence of sepsis in patients meeting two or more HLH criteria. Given the high mortality rates and the implied inflammatory activation in this group, an interconnection appears plausible. Fibrinogen is a key signaling molecule linking inflammation and coagulation, facilitating both physiological and pathological responses via different integrins like CD11b and GPIIb/IIIa [32]. While high fibrinogen levels may indicate a more controlled inflammatory response, decreased levels are frequently observed in critical illness due to hyperfibrinolysis, sepsis-induced coagulopathy, or excessive immune activation, as seen in HLH. Given that fibrinogen depletion can occur through multiple pathways, its association with mortality in our cohort likely reflects a combination of these factors rather than a single underlying mechanism. These findings suggest that fibrinogen assessment may help identify a distinct subset of critically ill patients with overlapping pathophysiologies, which conventional risk prediction models like the SAPS II score may not fully capture. While sCD25 and fibrinogen alone may not outperform these scores, their integration into future models could enhance predictive accuracy, particularly in terms of reflecting the interplay between inflammation and coagulation.

In contrast to the findings regarding sCD25 and fibrinogen, there are conflicting reports about ferritin as a dependable prognostic marker in the ICU setting. Although there is indisputable evidence regarding the diagnostic usefulness of ferritin for HLH and sepsis, its predictive value for mortality remains less clear [33]. In ICU patients with prolonged stays, ferritin dynamics often reflect the progression of organ dysfunction and closely align with the SOFA score rather than directly predicting survival [34]. While extreme ferritin elevations have been associated with adverse outcomes in HLH, there is no clear evidence supporting its prognostic value at admission. Our data suggest that ferritin may help detect hyperinflammatory states but lacks predictive power for survival in unselected critically ill patients. In contrast, sCD25 emerged as the strongest predictor in all our models, reinforcing its role as a key marker of immune activation beyond ferritin alone. Similar results were observed when assessing triglyceride levels as a standalone risk predictor for 30-day mortality. A previous publication emphasized the significance of various factors in influencing triglyceride levels, including age, insulin dosage, and hepatic failure. Moreover, evidence suggests a non-linear correlation between serum triglycerides and mortality in septic patients [35]. The reported U-shaped association may explain the lack of prognostic value when interpreting triglyceride levels as a continuous variable in our cohort. In this context, triglyceride levels can be compared to ferritin levels as crucial diagnostic factors for HLH, but display limited prognostic capabilities.

Finally, the increased mortality rates among patients meeting multiple HLH criteria underscore the importance of precise clinical monitoring, demonstrating the need for early diagnostic approaches and intensified resource allocation. The strong association of sCD25 and fibrinogen levels with survival emphasizes their significance as biomarkers in hyperinflammation and unselected ICU patients. Understanding their involvement in immunomodulating HLH, sepsis, and SIRS could shed some light on the underlying pathomechanisms and may unveil new therapeutic targets. Additionally, exploring the potential of incorporating sCD25, fibrinogen, and other markers into a combined risk model could improve predictive accuracy and help to create individualized treatment plans.

Our study has some limitations. First, the lack of an external validation cohort limits the direct transferability of our findings. Future multi-center studies are needed to validate these results in broader patient populations. However, the prospective all-comer design may serve as a strength, as it represents a diverse ICU patient population. Second, the observational nature of the study precludes conclusions about causality, and residual confounding factors cannot be ruled out entirely. Third, due to ethical and technical limitations, bone marrow biopsy and NK cell activity measurements were not performed, leading to an incomplete assessment of the HLH criteria. However, our approach focused on easily available HLH markers, with sCD25 included as a novel parameter in this setting, serving as an early indicator of HLH suspicion in critically ill patients. While additional criteria could further expand the inflammatory spectrum, it remains uncertain whether specific patient subgroups were missed due to the omission of two out of eight HLH criteria. Fourth, due to the extremely low prevalence of HLH, only one patient was detected in our cohort, with five positive HLH criteria according to the conventional definition. Nonetheless, our findings on sCD25 and fibrinogen may prove helpful in states of hyperinflammation and in unselected critically ill patients.

## 5. Conclusions

The present study highlights the prognostic significance of HLH criteria in critically ill patients. Individuals exhibiting two or more criteria at ICU admission experienced nearly a doubling in mortality as compared to patients with no or only one criterion met. The exceeding mortality rate of more than 40% in such patients underscores the need for early recognition and intervention in this population. Among the individual markers, sCD25 emerged as a significant predictor of survival, independently of traditional ICU risk factors. Furthermore, high fibrinogen levels were associated with improved outcomes and enhanced the predictive accuracy of the SAPS II score. Despite the established diagnostic relevance of ferritin and triglycerides in HLH, their prognostic value in predicting 30-day mortality was limited. Further research is essential to refine risk stratification and develop targeted therapies for HLH in the ICU and could ultimately improve patient outcomes.

## Figures and Tables

**Table 1:** Demographic and clinical baseline characteristics according to survival.

	Total *n* = 176 (100%)	30-Day Survivors *n* = 131 (74.4%)	30-Day Non-Survivors *n* = 45 (25.6%)	*p*-Value
***Patient characteristics:***				
Age [years]	67.5 (57.5–77.4)	65.8 (52.8–76.5)	71.0 (61.1–78.5)	0.218
Male sex, *n* (%)	108 (61.4%)	79 (60.3%)	29 (64.4%)	0.723
Vasopressors, *n* (%)	103 (58.5%)	69 (52.7%)	34 (75.6%)	**0.004**
Mechanical ventilation, *n* (%)	102 (58.0%)	71 (54.2%)	31 (68.9%)	**0.025**
C-reactive protein [mg/dL] Leukocytes max [G/L]	4.10 (1.02–10.67) 11.21 (7.69–16.17)	4.00 (0.89–10.68) 11.20 (7.69–16.43)	4.42 (1.53–10.42) 11.34 (7.71–16.11)	0.897 0.976
Fibrinogen [mg/dL]	392.0 (286.3–507.5)	416.0 (304.8–517.3)	358.0 (247.3–441.8)	**0.019**
Creatinine [mg/dL]	1.15 (0.87–1.85)	1.06 (0.86–1.68)	1.55 (1.11–2.70)	**<0.001**
Lactate [mmol/L]	1.9 (1.3–3.2)	1.8 (1.3–2.7)	2.7 (1.30–6.8)	**0.012**
Arterial pH	7.38 (7.28–7.48)	7.40 (7.30–7.48)	7.31 (7.22–7.48)	**0.036**
Bilirubin [mg/dL]	0.90 (0.60–1.40)	0.83 (0.52–1.22)	0.60 (1.05–1.80)	0.089
SIRS, *n* (%)	129 (73.3%)	90 (68.7%)	39 (86.7%)	**0.019**
SAPS II	45 (31–59)	39 (29–52)	61 (47–70)	**<0.001**
SOFA	8 (5–11)	7 (4–10)	12 (8–14)	**<0.001**
***Cause of admission:***				
Cardiac arrest, *n* (%)	40 (22.7%)	22 (16.8%)	18 (40.0%)	**<0.001**
ADHF, *n* (%)	16 (9.1%)	13 (9.9%)	3 (6.7%)	0.512
Cardiogenic shock, *n* (%)	25 (14.2%)	14 (10.7%)	11 (24.4%)	**0.023**
Sepsis/septic shock, *n* (%)	16 (9.1%)	12 (9.2%)	4 (8.9%)	0.956
Respiratory failure, *n* (%)	14 (8.0%)	10 (7.6%)	4 (8.9%)	0.788
Cardiac-surgery/valve intervention, *n* (%)	59 (33.5%)	55 (42.0%)	4 (8.9%)	**<0.001**
Other reasons, *n* (%)	6 (3.4%)	5 (3.8%)	1 (2.2%)	0.611

Categorial values are displayed as counts and percentages. Continuous values are shown as median and respective interquartile range (IQR) values. Statistical significance is illustrated in bold numbers. ADHF—acute decompensated heart failure, SAPS—simplified acute physiology score, SIRS—systemic inflammatory response syndrome, SOFA—sequential organ failure assessment. A total of 176 patients were evaluated in this analysis. *p*-values of <0.05 are considered statistically significant (Chi-square, Mann–Whitney test, ANOVA with Bonferroni correction, and unpaired Student’s *t*-test were used). Bold formatting was used to indicate statistical significance and to help the reader easily identify key parameters within the table.

**Table 2:** Demographic and clinical baseline characteristics according to HLH criteria.

	Total *n* = 176 (100%)	0–1 Criterion *n* = 147 (83.5%)	2+ Criteria *n* = 29 (16.5%)	*p*-Value
***Patient characteristics:***				
Age [years]	67.5 (57.5–77.4)	69.8 (58.8–77.5)	60.0 (48.6–72.1)	**0.031**
Male sex, *n* (%)	108 (61.4%)	92 (62.6%)	16 (55.2%)	0.454
BMI [kg/m^2^]	26.6 (23.9–30.1)	26.6 (24.0–30.9)	26.4 (21.3–29.7)	0.481
Heart rate [beats/min]	96 (81–111)	95 (81–110)	107 (88–120)	0.137
Mean blood pressure [mmHg]	64 (58–72)	65 (59–72)	65 (59–72)	0.148
Vasopressors, *n* (%)	103 (58.5%)	83 (56.5%)	19 (65.5%)	0.279
Mechanical ventilation, *n* (%)	102 (58.0%)	87 (59.2%)	15 (51.7%)	0.554
Hemoglobin [g/dL]	10.4 (9.3–11.8)	10.5 (9.5–11.9)	9.55 (8.3–10.28)	**0.001**
Thrombocytes [G/L]	171 (122–218)	184 (135–224)	98 (48–142.8)	**<0.001**
Leukocytes max [G/L]	11.21 (7.69–16.17)	11.83 (7.96–16.13)	10.05 (7.38–16.5)	0.678
C-reactive protein [mg/dL]	4.10 (1.02–10.67)	3.50 (0.89–10.07)	7.45 (3.85–20.63)	**0.003**
Fibrinogen [mg/dL]	392.0 (286.3–507.5)	396.5 (296.3–507.5)	380.5 (193.0–514.5)	0.436
Creatinine [mg/dL]	1.15 (0.87–1.85)	1.10 (0.86–1.68)	1.84 (1.04–2.95)	**0.006**
Lactate [mmol/L]	1.9 (1.3–3.2)	1.8 (1.2–3.1)	2.1 (1.5–3.7)	0.690
Arterial pH	7.38 (7.28–7.48)	7.38 (7.28–7.48)	7.36 (7.25–7.49)	0.489
Bilirubin [mg/dL]	0.90 (0.60–1.40)	0.83 (0.52–1.20)	1.59 (0.73–2.88)	**0.001**
SIRS, *n* (%)	129 (73.3%)	105 (71.4%)	24 (82.8%)	0.208
SAPS II	45 (31–59)	44 (30–57)	47.0 (37.5–60.0)	0.166
SOFA	8.0 (5.0–11.0)	7.0 (4.8–11.0)	10.0 (8.0–14.0)	**0.001**
***Cause of admission:***				
Cardiac arrest, *n* (%)	40 (22.7%)	35 (23.8%)	5 (17.2%)	0.440
ADHF, *n* (%)	16 (9.1%)	13 (8.8%)	3 (10.3%)	0.797
Cardiogenic shock, *n* (%)	25 (14.2%)	18 (12.2%)	7 (24.1%)	0.094
Sepsis/septic shock, *n* (%)	16 (9.1%)	10 (6.8%)	6 (20.7%)	**0.017**
Respiratory failure, *n* (%)	14 (8.0%)	10 (6.8%)	4 (13.8%)	0.204
Cardiac surgery/valve intervention, *n* (%)	59 (33.5%)	56 (38.1%)	3 (10.3%)	**0.004**
Other reasons, *n* (%)	6 (3.4%)	5 (3.4%)	1 (3.4%)	0.990

Categorial values are displayed as counts and percentages. Continuous values are shown as median and respective interquartile range (IQR) values. Statistical significance is illustrated in bold numbers. ADHF—acute decompensated heart failure, BMI—body mass index, SAPS—simplified acute physiology score, SIRS—systemic inflammatory response syndrome, SOFA—sequential organ failure assessment. A total of 176 patients were evaluated in this analysis. *p*-values of <0.05 are considered statistically significant (Chi-square, Mann–Whitney test, ANOVA with Bonferroni correction, and unpaired Student’s *t*-test were used). Bold formatting was used to indicate statistical significance and to help the reader easily identify key parameters within the table.

## Data Availability

The datasets used and/or analyzed during the current study are available from the corresponding author upon reasonable request.

## Supplementary Materials

The following supporting information can be downloaded at: https://www.mdpi.com/article/10.3390/jcm14061970/s1, Figure S1: No significant differences in mortality are present when comparing the cumulative survival of triglyceride tertiles during admission to the ICU (A). Furthermore, no differences are present when comparing tertiles of Ferritin (B); Figure S2: The combination of the SAPS II score (AUC 0.79) and sCD25 (AUC 0.69) results in an AUC of 0.81 (A). Moreover, the combination of the SAPS II score (AUC 0.79) and fibrinogen (AUC 0.62) results in an AUC of 0.83 (B); Figure S3: The SAPS II score and ferritin combination results in an AUC of 0.79 (A). Moreover, combining the SAPS II score and triglyceride levels results in an AUC of 0.80 (B); Table S1: Definition of HLH criteria; Table S2: Cox regression analyses for tertiles of sCD25 and HLH criteria.

## References

[1] van der Poll T., Shankar-Hari M., Wiersinga W.J. (2021). The immunology of sepsis. Immunity.

[2] Henter J.I., Samuelsson-Horne A., Arico M., Egeler R.M., Elinder G., Filipovich A.H., Gadner H., Imashuku S., Komp D., Ladisch S. (2002). Treatment of hemophagocytic lymphohistiocytosis with HLH-94 immunochemotherapy and bone marrow transplantation. Blood.

[3] Weitzman S. (2011). Approach to hemophagocytic syndromes. Hematol. Am. Soc. Hematol. Educ. Program..

[4] Bauchmuller K., Manson J.J., Tattersall R., Brown M., McNamara C., Singer M., Brett S.J. (2020). Haemophagocytic lymphohistiocytosis in adult critical care. J. Intensive Care Soc..

[5] Meena N.K., Sinokrot O., Duggal A., Alpat D., Singh Z.N., Coviello J.M., Li M., Wang X., Mireles-Cabodevila E. (2020). The Performance of Diagnostic Criteria for Hemophagocytic Lymphohistiocytosis in Critically Ill Patients. J. Intensive Care Med..

[6] Valade S., Monseau G., Mariotte E., Darmon M. (2021). Diagnostic Performance of Hemophagocytic Lymphohistiocytosis Criteria and HScore in Critically Ill Patients With Severe Hemophagocytic Syndrome. Crit. Care Med..

[7] Tothova Z., Berliner N. (2015). Hemophagocytic Syndrome and Critical Illness: New Insights into Diagnosis and Management. J. Intensive Care Med..

[8] Kumar G., Hererra M., Patel D., Nanchal R., Guddati A.K. (2020). Outcomes of adult critically ill patients with hemophagocytic lymphohistiocytosis in united states-analysis from an administrative database from 2007 to 2015. Am. J. Blood Res..

[9] Krychtiuk K.A., Ruhittel S., Hohensinner P.J., Koller L., Kaun C., Lenz M., Bauer B., Wutzlhofer L., Draxler D.F., Maurer G. (2015). Mitochondrial DNA and Toll-Like Receptor-9 Are Associated With Mortality in Critically Ill Patients. Crit. Care Med..

[10] Lenz M., Draxler D.F., Zhang C., Kassem M., Kastl S.P., Niessner A., Huber K., Wojta J., Heinz G., Speidl W.S. (2020). Toll-like receptor 2 and 9 expression on circulating neutrophils is associated with increased mortality in critically ill patients. Shock.

[11] Krychtiuk K.A., Honeder M.C., Lenz M., Maurer G., Wojta J., Heinz G., Huber K., Speidl W.S. (2017). Copeptin Predicts Mortality in Critically Ill Patients. PLoS ONE.

[12] Lenz M., Krychtiuk K.A., Goliasch G., Distelmaier K., Wojta J., Heinz G., Speidl W.S. (2020). N-terminal pro-brain natriuretic peptide and high-sensitivity troponin T exhibit additive prognostic value for the outcome of critically ill patients. Eur. Heart J. Acute Cardiovasc. Care.

[13] Henter J.I., Horne A., Arico M., Egeler R.M., Filipovich A.H., Imashuku S., Ladisch S., McClain K., Webb D., Winiarski J. (2007). HLH-2004: Diagnostic and therapeutic guidelines for hemophagocytic lymphohistiocytosis. Pediatr. Blood Cancer.

[14] Jordan M.B., Filipovich A.H. (2008). Hematopoietic cell transplantation for hemophagocytic lymphohistiocytosis: A journey of a thousand miles begins with a single (big) step. Bone Marrow Transplant..

[15] Chapman J., Goyal A., Azevedo A.M. StatPearls2023. https://www.ncbi.nlm.nih.gov/books/NBK430907/.

[16] Sjoberg B.P., Menias C.O., Lubner M.G., Mellnick V.M., Pickhardt P.J. (2018). Splenomegaly: A Combined Clinical and Radiologic Approach to the Differential Diagnosis. Gastroenterol. Clin. N. Am..

[17] Curovic Rotbain E., Lund Hansen D., Schaffalitzky de Muckadell O., Wibrand F., Meldgaard Lund A., Frederiksen H. (2017). Splenomegaly—Diagnostic validity, work-up, and underlying causes. PLoS ONE.

[18] Steinacher E., Lenz M., Krychtiuk K.A., Hengstenberg C., Huber K., Wojta J., Heinz G., Niessner A., Speidl W.S., Koller L. (2024). Decreased percentages of plasmacytoid dendritic cells predict survival in critically ill patients. J. Leukoc. Biol..

[19] DeLong E.R., DeLong D.M., Clarke-Pearson D.L. (1988). Comparing the areas under two or more correlated receiver operating characteristic curves: A nonparametric approach. Biometrics.

[20] Von Bahr Greenwood T., Palmkvist-Kaijser K., Chiang S.C., Tesi B., Bryceson Y.T., Hjelmqvist H., Henter J.-I. (2019). Elevated ferritin and soluble CD25 in critically ill patients are associated with parameters of (hyper) inflammation and lymphocyte cytotoxicity. Minerva Anestesiol..

[21] Huang C.M., Xu X.J., Qi W.Q., Ge Q.M. (2022). Prognostic significance of soluble CD25 in patients with sepsis: A prospective observational study. Clin. Chem. Lab. Med..

[22] Knaak C., Schuster F.S., Spies C., Vorderwulbecke G., Nyvlt P., Schenk T., Balzer F., La Rosée P., Janka G., Brunkhorst F.M. (2020). Hemophagocytic Lymphohistiocytosis in Critically Ill Patients. Shock.

[23] Cho E., Lee J.H., Lim H.J., Oh S.W., Jo S.K., Cho W.Y., Kim H., Lee S.Y. (2014). Soluble CD25 is increased in patients with sepsis-induced acute kidney injury. Nephrology.

[24] Bursa D., Bednarska A., Pihowicz A., Paciorek M., Horban A. (2021). Analysis of the occurrence of hemophagocytic lymphohistiocytosis (HLH) features in patients with sepsis: A prospective study. Sci. Rep..

[25] Jarczak D., Nierhaus A. (2022). Cytokine Storm-Definition, Causes, and Implications. Int. J. Mol. Sci..

[26] Fajgenbaum D.C., June C.H. (2020). Cytokine Storm. N. Engl. J. Med..

[27] Metcalf T.U., Wilkinson P.A., Cameron M.J., Ghneim K., Chiang C., Wertheimer A.M., Hiscott J.B., Nikolich-Zugich J., Haddad E.K. (2017). Human Monocyte Subsets Are Transcriptionally and Functionally Altered in Aging in Response to Pattern Recognition Receptor Agonists. J. Immunol..

[28] De Maeyer R.P.H., Chambers E.S. (2021). The impact of ageing on monocytes and macrophages. Immunol. Lett..

[29] Mori K., Tsujita Y., Yamane T., Eguchi Y. (2022). Decreasing Plasma Fibrinogen Levels in the Intensive Care Unit Are Associated with High Mortality Rates in Patients with Sepsis-Induced Coagulopathy. Clin. Appl. Thromb. Hemost..

[30] Yao C., Zhang G., Zhang N., Li R., Sun S., Zhang L., Xia Y., Chen S., Sun J., Chen M. (2023). Fibrinogen Is Associated with Prognosis of Critically Ill Patients with Sepsis: A Study Based on Cox Regression and Propensity Score Matching. Mediators Inflamm..

[31] Fricault P., Piot J., Estève C., Savan V., Sebesteyn A., Durand M., Chavanon O., Albaladejo P. (2022). Preoperative fibrinogen level and postcardiac surgery morbidity and mortality rates. Ann. Card. Anaesth..

[32] Davalos D., Akassoglou K. (2012). Fibrinogen as a key regulator of inflammation in disease. Semin. Immunopathol..

[33] Lachmann G., Knaak C., Vorderwulbecke G., La Rosee P., Balzer F., Schenk T., Schuster F.S., Nyvlt P., Janka G., Brunkhorst F.M. (2020). Hyperferritinemia in Critically Ill Patients. Crit. Care Med..

[34] Rusu D., Blaj M., Ristescu I., Patrascanu E., Gavril L., Lungu O., Siriopol I., Buzincu I., Grigoraș I. (2020). Outcome Predictive Value of Serum Ferritin in ICU Patients with Long ICU Stay. Medicina.

[35] Xiao M., Deng H., Mao W., Liu Y., Yang Q., Liu Y., Fan J., Li W., Liu D. (2023). U-shaped association between serum triglyceride levels and mortality among septic patients: An analysis based on the MIMIC-IV database. PLoS ONE.

