# Supplementary material for: Markers of Hemophagocytic Lymphohistiocytosis Are Associated with Mortality in Critically Ill Patients
Source: J Clin Med. 2025 Mar 14;14(6):1970. doi: 10.3390/jcm14061970 (PMC11943210; doi:10.3390/jcm14061970)
Supplement: Supplementary file 1 [file jcm-14-01970-s001.zip › jcm-3520661-supplementary.pdf]

Supplementary Table S1

Supplementary Table 1: Definition of HLH criteria.

- The presence of **fever**.
- The presence of **splenomegaly**.
- **Cytopenia** (at least two of three lineages in the peripheral blood):
  platelets <100 × 10^3/ml, haemoglobin <9 g/100 ml, neutrophils <1 × 10^3/ml.
- **Hypertriglyceridemia** (⩾265 mg/100 ml) and/or **hypofibrinogenemia** (⩽150 mg/100 ml).
- **Hemophagocytosis** in the bone marrow, spleen, or lymph nodes.
- Low or absent **NK cell activity**.
- **Ferritin** ⩾500 ng/ml.
- **sCD25** >2400 U/ml.

Supplementary Table S1. HLH; hemophagocytic lymphohistiocytosis, NK cells; natural killer cells, sCD25; soluble cluster of differentiation 25.

Supplementary Table S2

Supplementary Table 2. Cox regression analyses for tertiles of sCD25 and HLH criteria.

	Hazard ratio	95% CI	p-value
**Unadjusted**			
1st tertile sCD25	1	-	-
2nd tertile sCD25	1.12	0.46 – 2.76	0.804
3rd tertile sCD25	3.44	1.61 – 7.35	**0.001**
**Adjusted for age, sex, use of vasopressors and mechanical ventilation.**			
1st tertile sCD25	1	-	-
2nd tertile sCD25	0.97	0.40 – 2.48	0.976
3rd tertile sCD25	2.72	1.24 – 5.97	**0.012**
**Unadjusted**			
0–1 positive criterion of HLH	1	-	-
2 or more positive criteria of HLH	2.06	1.08 – 3.92	**0.029**
**Adjusted for age, sex, use of vasopressors and mechanical ventilation.**			
0–1 positive criterion of HLH	1	-	-
2 or more positive criteria of HLH	2.10	1.06 – 4.10	**0.033**
**Adjusted for age, sex, sepsis and mechanical ventilation.**			
0–1 positive criterion of HLH	1	-	-
2 or more positive criteria of HLH	2.07	1.06 – 4.02	**0.033**

Supplementary Table S2. HLH; hemophagocytic lymphohistiocytosis, sCD25; soluble cluster of differentiation 25. P-values of < 0.05 are considered statistically significant (Cox-proportional hazard regression analysis was used).

Supplementary Figure S1:

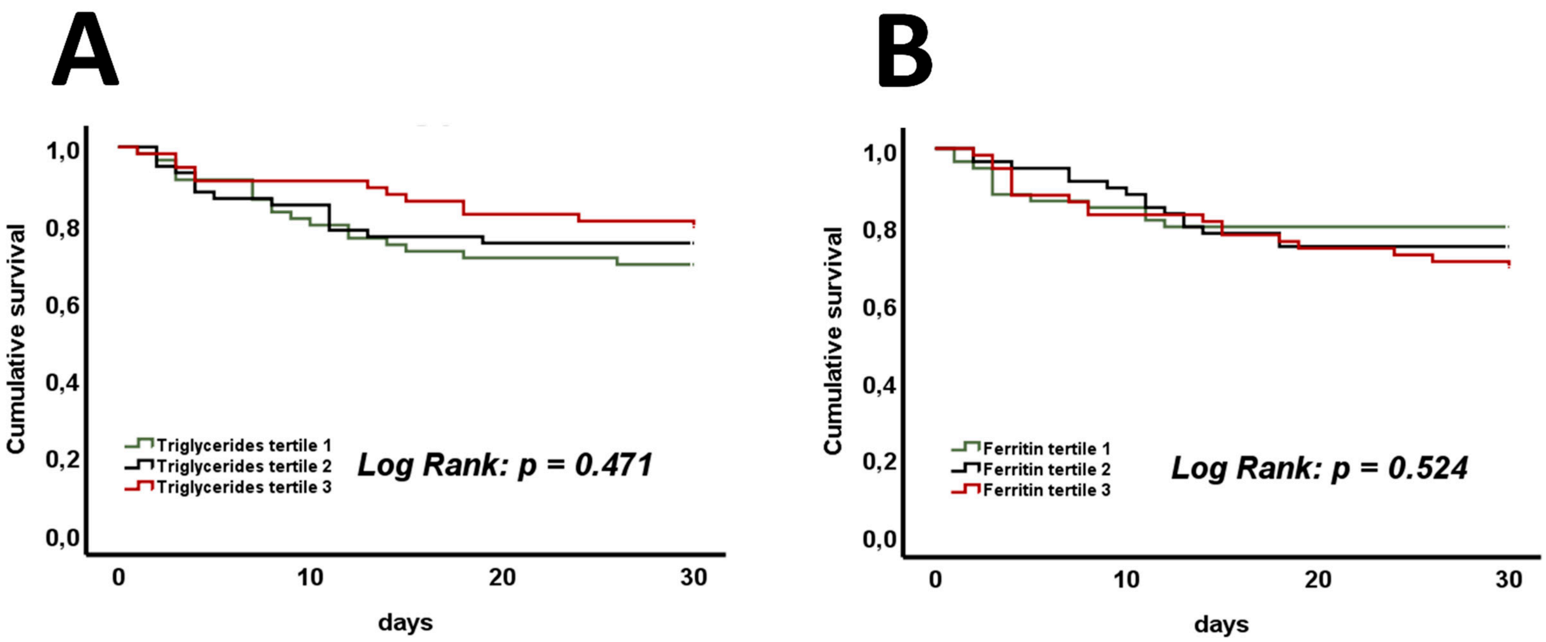

Suppl. Figure S1. No significant differences in mortality are present when comparing the cumulative survival of triglyceride tertiles during admission to the ICU (A). Furthermore, no differences are present when comparing tertiles of Ferritin (B). P-values of < 0.05 are considered statistically significant (log-rank test was used).

Supplementary Figure S2:

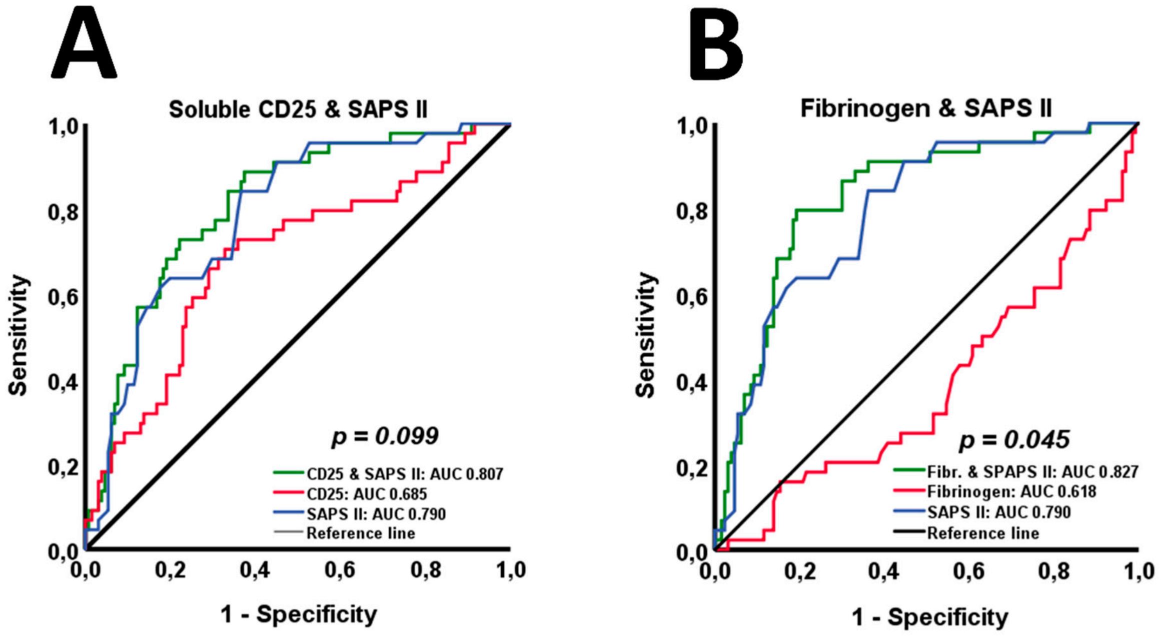

Suppl. Figure S2. The combination of the SAPS II score (AUC 0.79) and sCD25 (AUC 0.69) results in an AUC of 0.81 (A). Moreover, the combination of the SAPS II score (AUC 0.79) and fibrinogen (AUC 0.62) results in an AUC of 0.83 (B). P-values of < 0.05 are considered statistically significant (combined prognostic properties were calculated using logistic regression models). sCD25; soluble cluster of differentiation 25, SAPS II score; The Simplified Acute Physiology Score II, AUC; area under the curve.

Supplementary Figure S3:

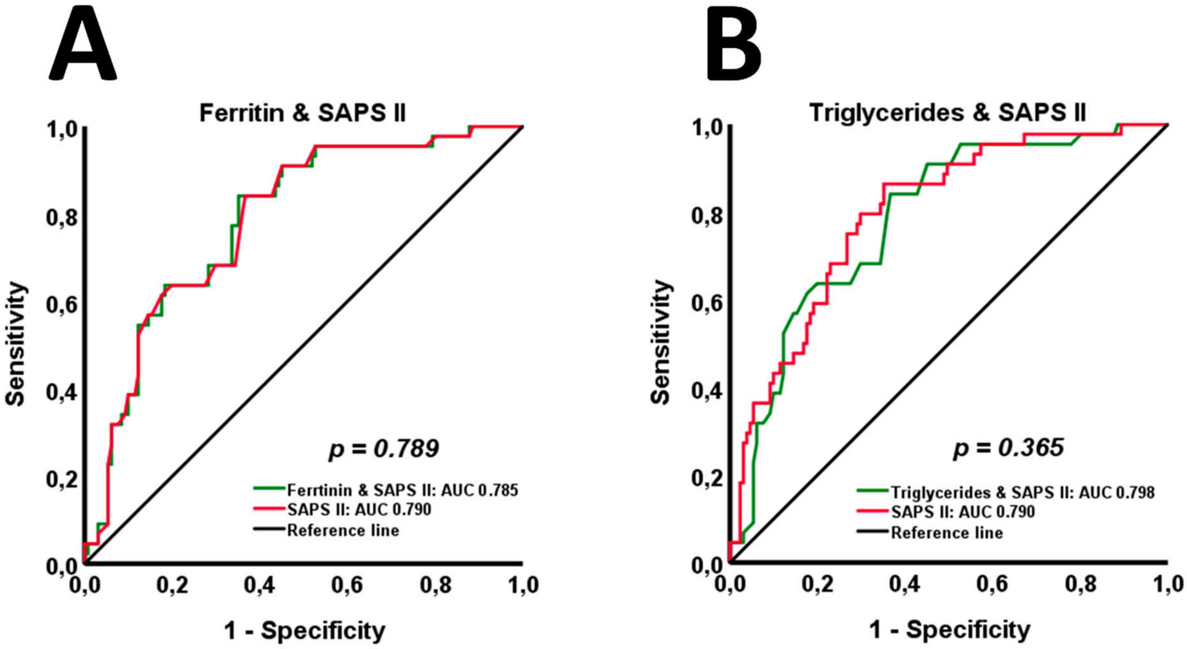

Suppl. Figure S3. The SAPS II score and ferritin combination results in an AUC of 0.79 (A). Moreover, combining the SAPS II score and triglyceride levels results in an AUC of 0.80 (B). P-values of < 0.05 are considered statistically significant (combined prognostic properties were calculated using logistic regression models). SAPS II score; The Simplified Acute Physiology Score II, AUC; area under the curve.
